# Fin modules: an evolutionary perspective on appendage disparity in basal vertebrates

**DOI:** 10.1186/s12915-017-0370-x

**Published:** 2017-04-27

**Authors:** Olivier Larouche, Miriam L. Zelditch, Richard Cloutier

**Affiliations:** 1 Laboratoire de Paléontologie et de Biologie évolutive, Université du Québec à Rimouski, Rimouski, Québec G5L 3A1 Canada; 2 Museum of Paleontology, University of Michigan, Ann Arbor, MI 48109 USA

**Keywords:** Fishes, Median fins, Paired fins, Morphological disparity, Phylogenetic supertree, Evolutionary modularity, Agnathans, Gnathostomes, Chondrichthyans, Osteichthyans

## Abstract

**Background:**

Fishes are extremely speciose and also highly disparate in their fin configurations, more specifically in the number of fins present as well as their structure, shape, and size. How they achieved this remarkable disparity is difficult to explain in the absence of any comprehensive overview of the evolutionary history of fish appendages. Fin modularity could provide an explanation for both the observed disparity in fin configurations and the sequential appearance of new fins. Modularity is considered as an important prerequisite for the evolvability of living systems, enabling individual modules to be optimized without interfering with others. Similarities in developmental patterns between some of the fins already suggest that they form developmental modules during ontogeny. At a macroevolutionary scale, these developmental modules could act as evolutionary units of change and contribute to the disparity in fin configurations. This study addresses fin disparity in a phylogenetic perspective, while focusing on the presence/absence and number of each of the median and paired fins.

**Results:**

Patterns of fin morphological disparity were assessed by mapping fin characters on a new phylogenetic supertree of fish orders. Among agnathans, disparity in fin configurations results from the sequential appearance of novel fins forming various combinations. Both median and paired fins would have appeared first as elongated ribbon-like structures, which were the precursors for more constricted appendages. Among chondrichthyans, disparity in fin configurations relates mostly to median fin losses. Among actinopterygians, fin disparity involves fin losses, the addition of novel fins (e.g., the adipose fin), and coordinated duplications of the dorsal and anal fins. Furthermore, some pairs of fins, notably the dorsal/anal and pectoral/pelvic fins, show non-independence in their character distribution, supporting expectations based on developmental and morphological evidence that these fin pairs form evolutionary modules.

**Conclusions:**

Our results suggest that the pectoral/pelvic fins and the dorsal/anal fins form two distinct evolutionary modules, and that the latter is nested within a more inclusive median fins module. Because the modularity hypotheses that we are testing are also supported by developmental and variational data, this constitutes a striking example linking developmental, variational, and evolutionary modules.

**Electronic supplementary material:**

The online version of this article (doi:10.1186/s12915-017-0370-x) contains supplementary material, which is available to authorized users.

## Background

**Fig. 1:**
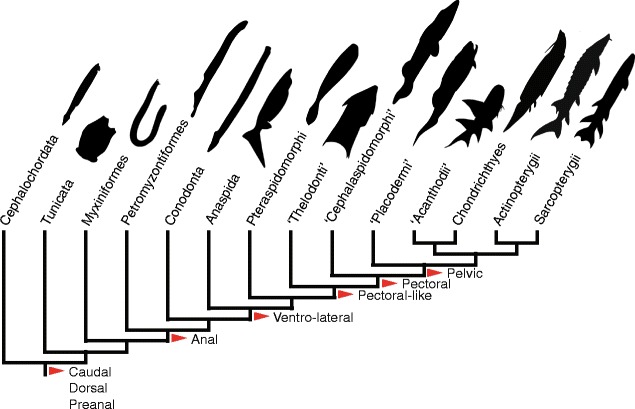
A sample of the disparity in fin configurations in extant and extinct fishes. The phylogenetic framework is a simplified version of the results of the supertree analysis (Fig. 2; Additional file 1: B). Nodes where new fins are sequentially added are identified

Fishes comprise the most basal representatives of the vertebrate lineage, a paraphyletic grouping that includes an astounding ~32,000 living species [1]. Fishes display a correspondingly high level of disparity in many aspects of their body plan (Fig. 1). For centuries, it has been recognized that part of this disparity is due to the numerous fin configurations, including the number of fins, their size, their position on the body, and their types of skeletal support [2–4]. Fins can be either median (dorsal, anal, caudal, and adipose fins) or paired appendages (pectoral and pelvic fins) that are used primarily for the purpose of locomotion. Morphological disparity in fin configurations of living fishes can readily be observed when considering the presence or absence of these appendages: examples of fin losses are known for each of the median and paired fins, including the caudal fin (e.g., *Mola mola*). Alternatively, fins can also be duplicated or even triplicated (e.g., the dorsal fins in *Gadus morhua*). In some cases entirely new fins can emerge, as in the case of the adipose fin in some teleosts [5].

The evolutionary sequence leading to the origin of fish appendages has not been completely resolved yet (although a good synthesis of what is known at the molecular level is provided in [6]). It is generally acknowledged that fins first appeared as median dorsal and ventral structures during the Lower Cambrian (ca. 535 Ma): the oldest known vertebrate fossils display well-developed median fins but no paired fins [7–9]. The anaspids, a group of jawless fishes, are the most primitive known vertebrates with unambiguous paired fins [10]. However, the most basal vertebrates that conclusively display endoskeletal structures and associated musculature in paired appendages are among another group of jawless fishes, the osteostracans [10–12]. The osteostracan paired fins are considered by most as homologous to the pectoral fins of jawed vertebrates (e.g., [11, 13–15]), while the pelvic fins appeared later on among stem gnathostomes [16–19]. Thus, the fossil record seemingly indicates that the pectoral fins appeared before the pelvic fins [10, 20, 21].

One potential explanation for both the emergence of new fins and the observed disparity in fin configurations is that fins are modular. Modularity, defined most broadly, means that organisms can be decomposed into smaller components which are termed modules [22–24]. Modules are therefore discrete and internally coherent units that may develop and also evolve quasi-independently from other modules [25–27]. There are different kinds of modules that are defined according to the processes in which they are involved. Developmental modules are parts of an organism that are quasi-autonomous in their patterns of formation and differentiation [28–30], variational modules comprise traits that covary within populations [30–32], and evolutionary modules comprise traits that co-evolve [31, 33, 34]. Because of their quasi-independence, developmental modules may correspond to variational or evolutionary modules as well [26, 34]. Modules may also be susceptible to duplication, dissociation, divergence, and/or co-option [28]. This can lead to the repetition of individual structures that, if decoupled, can subsequently follow their own evolutionary trajectories [23, 28, 35]. Such a process of modular duplication followed by decoupling has been hypothesized to facilitate the emergence of morphological and/or functional innovations [23, 28, 36–38]. Thus, the concept of modularity is well suited to investigate the functional and developmental disparity observed in organisms [30], and it may account for the high disparity of fin configurations in fishes.

Hypotheses of modularity have already been proposed for both median and paired fins in fishes. Four fundamental modules involved in the positioning and patterning of median fins have been hypothesized for living actinopterygians (ray-finned fishes): the designated positioning modules refer to similar positions along the body axis between the dorsal and anal fins, while the designated patterning modules refer to similarities in anatomical development between these two fins [39]. Some of these patterning modules have also been identified in fossil actinopterygians and sarcopterygians (lobe-finned fishes), suggesting that they could have been inherited from a common ancestor to all osteichthyans (bony fishes, comprising all actinopterygians and sarcopterygians) [40]. Developmental evidence from the catshark (*Scyliorhinus canicula*) also indicates that modules might be involved in the positioning of the dorsal and anal fins in chondrichthyans (cartilaginous fishes) [41]. It has also been suggested that developmental mechanisms could have been co-opted from the median fins, leading to the emergence of the paired fins [6, 41–44]. Furthermore, most authors generally consider that the pectoral fins appeared before the pelvic fins [10, 13, 17, 20, 21], leading to the hypothesis that the pelvic fins might represent a reiteration of the pectoral fins module. Duplication of a paired fins module followed by decoupling would help to explain why pectoral and pelvic fins can be altered or lost independently [10, 45, 46].

Modularity can offer a valuable framework to investigate both the emergence of morphological disparity in fin configurations and the sequential appearance of fins in vertebrates. New fins could arise through processes such as fin module duplications that could subsequently become decoupled on an evolutionary timescale. However, two main issues complicate the interpretation of these evolutionary transformations of fins: (1) the lack of consensus regarding the homology of structures found in early vertebrate appendages [47] and (2) the lack of consensus concerning phylogenetic relationships among fishes (see, e.g., [12, 48–61]). With this in mind, this study has two principal objectives. The first is to characterize the morphological disparity in fin configurations in an evolutionary perspective, and to investigate possible scenarios for the sequential appearance of each one of the median and paired fins. This evolutionary perspective required a phylogenetic context for the analyses. Because a complete phylogeny of extinct and extant fishes at the ordinal level has not yet been published, we used a supertree approach to summarize findings from recent investigations of basal vertebrate interrelationships. The second objective is to analyze covariation patterns between fins in terms of their presence/absence at a macroevolutionary scale, which could indicate their evolutionary modularity. We predicted that fins that are hypothesized to share developmental or evolutionary modules controlling their positioning and/or patterning should also covary in their presence/absence data.

## Methods

### Fin presence/absence dataset

#### Taxonomic selection

A dataset was constructed using a sample of representative species from 144 orders of fishes. Ordinal classification of extant species followed Nelson et al. [1] and Fishbase [62]. For fossil taxa, ordinal classifications of different authors have been used in relation to the taxonomic group, with some minor taxonomic modification based on the most recent literature: Janvier [16] for agnathans in general; Märss et al. [63] for thelodonts; Denison [64] and Young [65] for placoderms; Nelson et al. [1] for acanthodians; Ginter et al. [66] for elasmobranchs and Stahl [67] for holocephalians; Nelson et al. [1] for actinopterygians; and Cloutier and Ahlberg [68] for sarcopterygians. The sample comprised a total of 2730 taxa (607 extinct, 2123 extant), representing about 9% of current estimates of fish species richness (~32,000 living species [1]). Species were selected to maximize diversity within each one of the orders. This was done by sampling individual orders proportionately to their species richness while maximizing the number of families and genera taken into account. Special care was taken not to oversample extreme morphologies. For the fossil data, a selection was made based on the availability and completeness of morphological data. Furthermore, scoring of fin characters of fossil taxa was based mostly on photographs and on the descriptive work, and not only on published paleontological reconstructions. The inclusion of fossil taxa is important to reveal basal character states which might not be observable in more recent and derived forms.

#### Appendage terminology

The extensive disparity in fin morphologies and the debated homologies of some of the fins among different groups of fishes required that we come up with consistent defining criteria for each of them. We did not define the fins based on strict homology criteria in order to be able to score for the totality of the disparity encountered in the analysis. Patterson [69] proposed that three criteria should be used to define homologous structures: (1) similarity (topographical correspondence and ontogenetic transformation), (2) conjunction (or anatomical singularity), and (3) congruence (phylogenetic congruence with other homologies). In our case, the identity of the fins was established largely on the basis of a positional criterion, which is one of these criteria (topographical similarity) used to assess homology between structures [69]. Structural and ontogenetic criteria were also used when position alone was insufficient to clearly define some fins and when these data were available from the literature. The definitions used for the scoring of fin characters are provided in Table 1.

**Table 1:** Terminology used to define fins for the scoring of characters among taxa

Terms used in this paper	Definition	Other terms that have been used
Median ventral fin	An unpaired ventral finfold that can be inserted either anteriorly (e.g., some Myxiniformes) or posteriorly to the anus, and anteriorly to the anal fin when it is present (e.g., some Stomiidae, Paralepididae and Phallostethidae (Teleostei))	Preanal finfold (or skinfold), ventral adipose fin
Ventrolateral paired fins	Ventrolaterally positioned fins or fin supports placed along the trunk that are generally long-based and that are of uncertain homology to the pectoral and/or pelvic fins	Ventrolateral finfolds, intermediate spines, prepelvic spines
Pectoral fins	Short-based paired fins inserted on the thorax close to the gill openings	Suprabranchial fins, paired flaps, pectoral flaps, pectoral swimming appendages
Pelvic fins	Ventrally inserted short-based paired fins, always located anteriorly to the anus/cloaca	Ventral fins
Dorsal fin	Fins located on the dorsal midline of the body, between the head and the tail	
Anal fin	Fins located on the ventral midline between the anus (or cloaca) and the tail	
Adipose fin	A small non-rayed fin usually located medially between the dorsal and caudal fins; this median fin is present among several groups of ostariophysans and basal euteleosts	Fatty fin, dorsal organ, dorsal filament
Caudal fin	The caudal fin is located at the extremity of the tail	Tail fin

#### Appendage coding

Species considered in the analyses were scored for the presence or absence and the number of each one of the fins: dorsal fin (0, 1, 2, or 3), adipose fin (0 or 1), median ventral fin (0 or 1), anal fin (0, 1, or 2), caudal fin (0 or 1), ventrolateral paired fins (0 or 1), pectoral fins (0 or 1) and pelvic fins (0 or 1). The scoring reflected the number of each fin present and had no implications as to the plesiomorphic or apomorphic condition of characters. Presence or absence of each of the fins was assessed based on multiple sources including specimen descriptions, photographic material, radiographs, illustrations, and paleontological reconstructions.

### Fish supertree

Our investigation of fin morphological disparity required a phylogenetic framework. Yet, phylogenies of basal vertebrates have not reached a generalized consensus (see, e.g., [12, 48–61]), and a complete phylogeny of fossil and extant fishes at the ordinal level that encompasses the entire taxonomical span considered in this study is not currently available.

The phylogenetic framework was constructed using a supertree approach, more specifically with the matrix representation with parsimony (MRP) algorithm [70–72], which is the most commonly used method [73–76]. The MRP method is well suited for inferring topologies from diverse partially overlapping datasets [70–72, 77, 78], notably for the joint analysis of fossil and extant data [74, 79]. A set of 118 source trees (Additional file 1: A) was compiled. Selected source trees were required to have been generated using modern computer-based phylogenetic analyses (the analyses used were published between 1986 and September 2016) of either morphological or molecular datasets. The supertree was constructed using the phangorn package [80] in R version 3.2.4 [81]. Source trees were simplified at the ordinal level whenever necessary. Some fish orders continue to be used for taxonomic simplification even though they are most likely paraphyletic or polyphyletic (e.g., Climatiiformes, Perciformes, Osteolepiformes). These orders were subdivided into smaller units that could be assigned to multiple nodes in individual source trees prior to generating the consensus topology. Phylogenetic supertrees were reconstructed with the maximum parsimony function that generates a single most parsimonious solution, and with Nixon [82]’s Parsimony Ratchet that performs heuristic searches and generates a set of most parsimonious trees [80].

In order to manage the broad phylogenetic scope of the source trees, five separate supertree analyses were conducted, i.e., agnathans, basal gnathostomes (placoderms and acanthodians), chondrichthyans, actinopterygians, and sarcopterygians. The interrelationships among these larger more inclusive groups are well resolved, and the resulting trees from each individual analysis were thus combined to generate the complete supertree of fishes. An exception to this concerns the interrelationships between placoderms, acanthodians, and crown gnathostomes, which are currently strongly debated. For this purpose, the supertree analysis focusing on these stem gnathostome groups incorporated chondrichthyans and osteichthyans as terminal branches.

The MRP supertree method combines source trees with the assumption that datasets are independent [73, 76, 83, 84]. However, in this case some source trees cannot be considered as independent, particularly for the fossil groups where phylogenetic analyses often build upon previously published data matrices, adding or re-scoring taxa and characters. To reduce this bias, care was taken to select source trees where either the character sets or the list of taxa had been substantially modified. The impracticability of this assumption of total independence of source trees is a well-known issue in supertree constructions, and non-independence is unlikely to be completely eliminated [74, 76, 83, 85–88].

Phylogenetic supertrees have also been criticized for losing contact with the primary data from which they are derived [84, 85, 88–91]. Bryant ([84], p. 366) added that MRP supertrees are likely to violate at least some phylogenetic principles and are consequently best considered as a “heuristic synthesis of available hierarchical information, rather than the products of rigorous phylogenetic analysis.” Nonetheless, phylogenetic supertrees provide a reasonable alternative to an analysis based on total evidence in situations where such an approach is unavailable [71, 73, 74].

### Mapping of the fin characters on the supertree

**Fig. 2:**
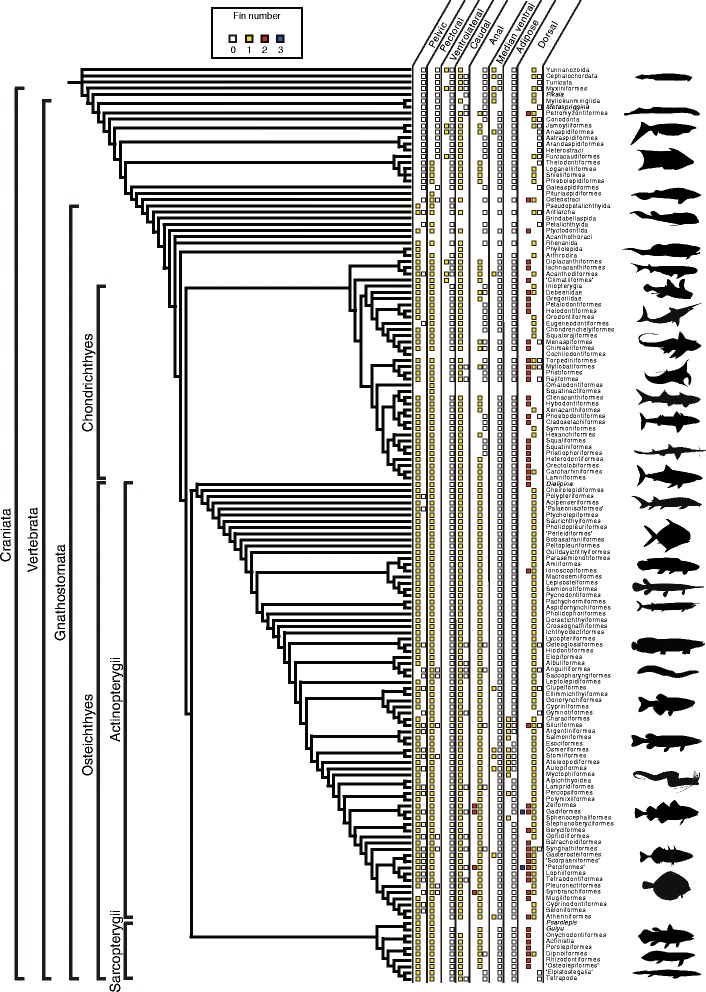
Distribution of fin conditions on the supertree of fishes showing the interrelationships among extant and extinct orders of fishes. Some non-ordinal ranked taxa that were included in the supertree analysis have been pruned for simplification. *Colored squares* above terminal branches represent sampled fin character states for each order/subgroup

A summary of the presence/absence data was mapped on the supertree (Fig. 2). This was done by compiling the observed character states for individual fins within each of the orders (Additional file 2), and mapping the information on the terminal branches of the tree. This allowed us to explore possible scenarios of sequential appearance of fins, as well as to determine where most of the morphological disparity in fin configurations was concentrated in the phylogeny of fishes.

### Covariation in the number of fins present

Multiple correspondence analyses were used as an exploratory method to investigate covariation among fins in the presence/absence data. Relationships between pairs of fins were also statistically tested using Fisher’s exact test. Since Fisher’s exact test only tells us if there is non-independence in frequencies of observations between two qualitative variables, we used Spearman’s rank correlation coefficients to establish the direction of the relationships between variables. For each pair of fins, a positive correlation indicates that they tend to be jointly present or jointly absent. Alternatively, a negative correlation means that the variables have opposite trends: for instance, one fin is present while the other is absent.

As described in the previous section, prior to running the analyses, the dataset was summarized by identifying all of the unique fin combinations within individual orders. An analysis was also performed by identifying all unique combinations for the entire dataset, excluding combinations that contained missing data, resulting in 51 fin combinations. Sensitivity analyses were performed to assess the effect of missing data: this was done by running separate analyses where taxa with the most missing fin characters were sequentially removed.

Multiple correspondence analyses were performed using the ca package [92], and Spearman’s rank correlations were calculated with the Hmisc package [93] in R version 3.2.4 [81].

## Results

### Fish supertree

The phylogenetic supertree analysis summarizes the topologies of 17 source trees for agnathans, 13 trees for basal gnathostomes (placoderms and acanthodians), 24 trees for chondrichthyans, 39 trees for actinopterygians, and 25 trees for sarcopterygians. The supertree generated using Nixon’s Parsimony Ratchet and 50% majority rule consensus contains 163 terminal branches and 156 internal nodes, making it 96.3% resolved compared to a fully dichotomous phylogenetic tree. The strict consensus tree contains 142 internal nodes, making it 87.6% resolved. The single most parsimonious solution using the optimum parsimony setting generated a tree with 160 internal nodes, making it 98.8% resolved. We used a pruned version of the 50% majority rule supertree (Additional file 1: B) for the mapping of fin characters (Fig. 2). The strict consensus and the optimum parsimony solutions can be found in Additional file 1: C, D.

To our knowledge, this is the first time an attempt has been made to reconcile such a large number of fish orders within a single supertree using modern phylogenetic methods. Most recently published trees with broad taxonomic scopes have focused on interrelationships among agnathans, basal gnathostomes, or derived actinopterygians (Percomorpha). The phylogenetic relationships of basal vertebrates, particularly the interrelationships of fossil taxa, have been debated for many years (Additional file 1: E). Among these contentious groups, our consensus topology, generated from recent phylogenetic analyses, posits that living agnathans are paraphyletic, thelodonts are monophyletic, placoderms are stem gnathostomes, and acanthodians are stem chondrichthyans. As for chondrichthyans, actinopterygians, and sarcopterygians, the supertree analysis recovered most ordinal groupings that have generally been recognized as clades (e.g., Euchondrocephali, Elasmobranchii, Squalomorphii, Galeomorphii, Osteoglossomorpha, Elopomorpha, Otocephala, Acanthomorpha, Tetrapodomorpha).

### Mapping the evolutionary history of fish appendages

The mapping of the presence/absence data on the supertree (Fig. 2) allows us to (1) visually establish where most of the morphological disparity occurs within the phylogeny and (2) infer the order of appearance of each of the fins. There are three sections of the phylogeny where most of the disparity in fin configurations is concentrated: (1) agnathans, (2) chondrichthyans, and (3) derived actinopterygians. The disparity in agnathans is largely due to the original diversification of fin configurations and the sequential addition of novel fins within this paraphyletic assemblage of basal fishes, while the disparity in chondrichthyans and actinopterygians results mostly from fin losses, duplications of pre-existing fins, or the addition of new fins.

#### Agnathans

With the exception of the pelvic and adipose fins, most fins appear early during the evolutionary history of fishes. Median fins are already present along the dorsal and ventral midline, even in the most basal craniates and vertebrates (e.g., *Haikouella*, *Myllokunmingia*, and *Haikouichthys*). The fin which extends along the ventral midline in these forms is positioned anteriorly to the anus and as such does not qualify as an anal fin. This fin configuration is reminiscent of what is observed in cephalochordates, although in the latter case, the dorsal finfold is comparatively longer in its anterior extent, reaching the tip of the notochord where it forms the “rostral fin.” In *Haikouella* and *Haikouichthys*, the median fins are continuous around the tail, where they form a rudimentary caudal fin. A well-developed caudal fin is a generalized feature of all other agnathan taxa with the exception of a few hagfishes and lampreys. The presence of a caudal fin early in the evolutionary history of fishes is expected, considering that a postanal tail is a chordate synapomorphy: even tunicates possess a caudal fin prior to metamorphosis [94]. An anal fin is present in a few Carboniferous lampreys and is commonly found in anaspids and some thelodonts.

Paired fins also arise among agnathans. Paired fins first occur as long-based ribbon-like fins with parallel support structures, such as in anaspids. In osteostracans and some thelodonts, the paired fins are shorter-based and have a position comparable to that of pectoral fins. However, although homology of the osteostracan paired fins to the gnathostome pectoral fins has been a generalized view for many years, most authors remain conservative in such an interpretation for the thelodont paired fins. Indeed, many authors acknowledge the similarity in positioning but avoid making a statement of homology by referring to these fins as “pectoral-level fins,” “pectoral appendages,” or as “suprabranchial fins” (see, e.g., [11, 47, 50, 95–98]).

The disparity in fin configurations within agnathans is predominantly due to differences among taxa as to the presence or absence of the median and paired fins as they successively appear during the evolutionary history of these early fishes, generating novel combinations in different groups. A few taxa also possess two dorsal fins, a condition that seems to have evolved independently in lampreys and osteostracans. Based on the number of character changes on the supertree, the presence/absence of the dorsal and anal fins seem to be the most important source of disparity in fin configurations in agnathans, followed by the median ventral fin and ventrolateral paired fins.

#### Chondrichthyans

In chondrichthyans, some fins contribute very little to the disparity in fin configurations: the pectoral fins are always present, the pelvic fins are lost only in the Eugeneodontiformes, and the caudal fin is always present, except in Myliobatiformes, where it is generally absent, and in Rajiformes, where it is occasionally absent. Most of the disparity in fin configurations relates to the dorsal and anal fins. The majority of chondrichthyans have one or two dorsal fins, although some forms are characterized by the absence of this fin (e.g., some Rajiformes, Torpediniformes, and Myliobatiformes). Absence of the anal fin is much more frequent than that of the dorsal fin, particularly among batoids, squalomorphs, and holocephalans: the anal fin is lacking in at least some representatives of 18 chondrichthyan orders, whereas the dorsal fin is lacking in some representatives of only five orders.

#### Actinopterygians

Basal actinopterygians show very little disparity in their fin configurations: they generally have single dorsal, anal, and caudal fins, and paired pectoral fins. The only source of disparity concerns the occasional loss of the pelvic fins and the presence of two dorsal fins in *Dialipina salgueiroensis* [99] and *Placidichthys bidorsalis* [100]. The second dorsal fin in *Dialipina* supports the hypothesis that the plesiomorphic condition for basal gnathostomes might have been the presence of two dorsal fins [16, 99, 101, 102], whereas the presence of two dorsal fins in *Placidichthys* is secondarily derived.

In contrast, derived actinopterygians are much more disparate in their fin configurations. Part of this disparity can be accounted for by repeated losses of some of the fins. Indeed, any of the median and paired fins, including the caudal fin, can be lacking in some actinopterygian groups. The pelvic fins are the most frequently lost: they are reported as absent in representatives of 26 orders from our dataset. Comparatively, only 11 orders contain species that lack a caudal fin, eight orders contain species that lack pectoral fins, six orders contain species that lack the dorsal fin, and five orders contain species that lack an anal fin. The disparity among derived actinopterygians can also be explained partly by apparent duplications of the median fins. Derived actinopterygians, more specifically the Acanthomorpha, frequently possess more than one dorsal and/or anal fin: 13 actinopterygian orders contain species that have at least two separate dorsal fins, and four of these orders contain species that also have two anal fins. Furthermore, some species of two acanthomorph orders even show three separate dorsal fins. Yet another source of disparity in actinopterygians is brought about by the addition of novel fins. The adipose fin is considered as such an evolutionary novelty [5, 103] which first appears among the Ostariophysi. An adipose fin is present in representatives of 11 actinopterygian orders from our dataset. In some of these groups, it is occasionally combined with a median ventral fin positioned anteriorly to the anal fin (referred to as a ventral adipose fin in morphological descriptions), although this ventral fin can be present even if the dorsal adipose fin is absent. A median ventral fin is found in some species of six of the orders sampled in our actinopterygian dataset.

#### Sarcopterygians

Sarcopterygians display far less disparity in their fin configurations: differences among taxa are limited to the number of dorsal fins and the presence/absence of the dorsal, anal, and caudal fins. Furthermore, only two orders (Dipnoiformes and “Elpistostegalia”) show some disparity in fin configurations among the species they contain. Most sarcopterygians have two dorsal fins, a single anal fin, a caudal fin, and paired pectoral and pelvic fins. The Dipnoiformes are the most disparate order in terms of fin configurations: there can be either one or two dorsal fins, and a separate anal fin is lost in derived dipnoans. Elpistostegalians, the most derived piscine tetrapodomorphs, are characterized by the loss of the dorsal and anal fins, although the caudal fin remains. The paired fins are conserved in all sarcopterygian taxa, including tetrapods where they evolved towards the forelimbs and hindlimbs [10, 16, 17, 45, 104–108].

### Covariation in the presence/absence of fish appendages

**Fig. 3:**
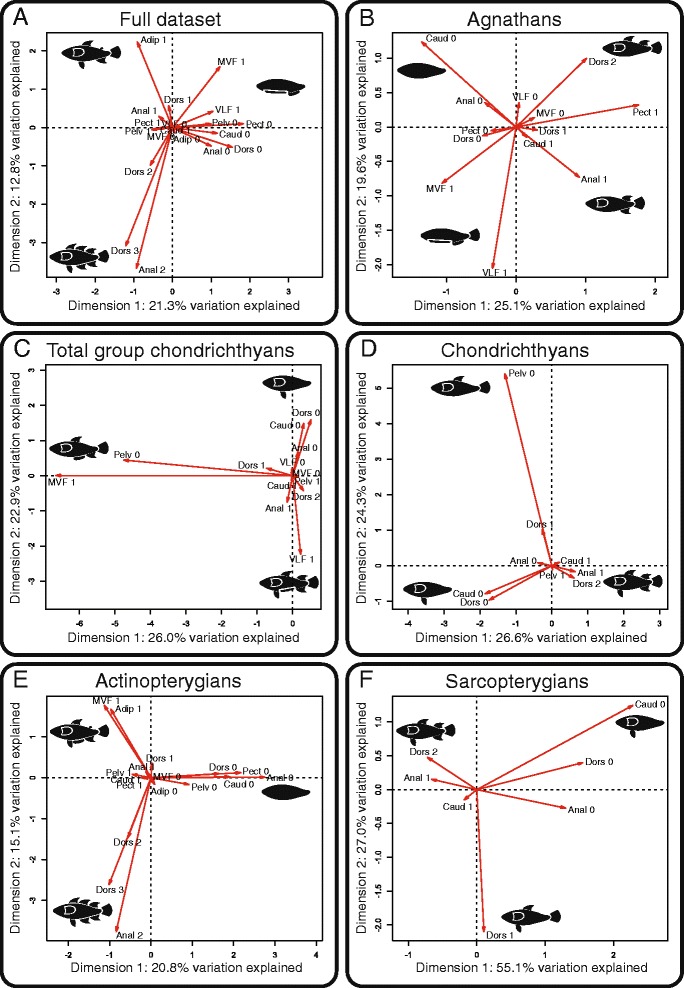
Biplots of the multiple correspondence analyses. *Lettered panels* represent the results for **a** the entire dataset, **b** agnathans, **c** total group chondrichthyans, **d** chondrichthyans, **e** actinopterygians, and **f** sarcopterygians. *Fish silhouettes* represent the major trends in fin configurations among each group

Multiple correspondence analyses were performed with the complete dataset (147 orders) and also on six subsets of the data corresponding to major taxonomic groups (i.e., agnathans (18 orders), total group chondrichthyans (37 orders), undoubted chondrichthyans (excluding acanthodian-like taxa; 33 orders), actinopterygians (73 orders), and sarcopterygians (7 orders)), most of which are monophyletic with the exception of agnathans. We found that the removal of taxa with missing data was for the most part inconsequential, affecting only the percentage of variance explained by the major axes of variation. As such, we focus on the results incorporating the entire dataset (Fig. 3). We limited our analysis to the first two dimensions of the MCAs because additional dimensions showed increasingly rare fin combinations in the dataset and were not biologically interpretable.

The first two dimensions of the MCA on the entire dataset (Fig. 3a) explain 21.3 and 12.8% of the variation, respectively. The first dimension contrasts fishes bearing only median ventral fins and/or ventrolateral paired fins, to fishes that have two or three dorsal and anal fins, or an adipose fin. This can be interpreted as contrasting patterns of fin configurations found in basal agnathans to those of acanthomorphs. The second dimension contrasts fishes bearing an adipose fin and a median ventral fin to those that have two (or three) dorsal and anal fins. An adipose fin is commonly found in a number of orders of basal euteleosteans, whereas the presence of three dorsal fins and two anal fins is only found in the more advanced euteleosteans (Acanthomorpha). Additionally, based on the acute angles between some vectors and similarities in their relative lengths, there is evidence for coordinated losses or duplications between some of the fins. This is observed for the coordinated duplications (or triplications) of the dorsal and anal fins, as well as for the coordinated losses of the dorsal and anal fins, and of the pectoral and pelvic fins.

The first two dimensions of the MCA on agnathans (Fig. 3b) explain 25.1 and 19.6% of the variation, respectively. The first dimension contrasts fishes that have caudal, dorsal, anal, and pectoral fins to those that do not have these fins. The former pattern is characteristic of fishes close to the agnathan-gnathostome transition, such as osteostracans, while the latter fin configuration is found in the more basal agnathans that have long-based ribbon-like median and paired fins. The second dimension contrasts primarily a morphology where most fins are absent with the exception of two dorsal fins, a pattern found in a single petromyzontid fossil species, to forms where there is a single copy of each fin. The angles between the vectors for each of the fins are relatively equal, resulting in a star-shaped pattern: this suggests that there is little covariation among the fins and that they are all independent from one another. Alternatively, this star-shaped pattern could also partly result from the difficulties in correctly identifying homologies in fin characters among agnathans. It has been suggested, for example, that the paired fins of some anaspids and thelodonts could be homologous to the pelvic fins of gnathostomes [109].

For the total group chondrichthyans (i.e., including acanthodians and putative chondrichthyans) (Fig. 3c), the first two dimensions of the MCA explain 26.0 and 22.9% of the variation, respectively. The first dimension contrasts forms that have a single dorsal fin, no pelvic fins, and a median ventral fin, to fishes that have lost their median fins and/or have two dorsal fins. This can be interpreted as opposing the morphology of a single genus of acanthodians, *Acanthodes*, to the disparity in batoid morphologies. The second dimension opposes forms that have ventrolateral paired fins, an anal fin, and two dorsal fins, to forms that have lost their median fins. This opposes a morphology found in climatiid and diplacanthid acanthodians to the loss of median fins found in some batoids, more specifically among the Rajiformes and Myliobatiformes. As with the analysis on the full dataset, the relative lengths and angles between some of the vectors show coordinated patterns between some of the fins, in this case suggesting coordinated losses of the dorsal, anal, and caudal fins.

In the analysis restricted to undoubted chondrichthyans (Fig. 3d), the first two dimensions of the MCA explain 26.6% and 24.3% of the variation, respectively. The first dimension contrasts forms that have lost most of their median fins and the pelvic fins, to morphologies where all of the fins are present including two dorsal fins. The latter pattern is characteristic of the non-batoid neoselachians (Galeomorphii and Squalomorphii), while the former seems to be a combination of characters found either in batoids or in the Eugeneodontiformes. The second axis primarily contrasts forms that have lost the pelvic fins and have a single dorsal fin to those that have lost the dorsal and caudal fins or have two dorsal fins. As such, this dimension of the MCA opposes the Eugeneodontiformes, the only chondrichthyan taxon where the pelvic fins are absent, to the disparity patterns of other chondrichhyans.

In actinopterygians, the first two dimensions of the MCA (Fig. 3e) explain 20.8 and 15.1% of the total variation, respectively. The first dimension contrasts forms that have lost all of their fins to morphologies with additional fins. These accessory fins correspond either to the addition of second and third dorsal and anal fins, or to the addition of median ventral fins and adipose fins. The loss of all fins is rare and can be found only in a few Anguilliformes and Gasterosteiformes, generally associated with an elongated body shape. The pattern with supplementary fins is characteristic of the more advanced teleosteans (Ostariophysi and Euteleostei). The second dimension opposes forms where a median ventral fin and an adipose fin are present to forms with serial duplications of the dorsal and anal fins. This contrasts two ways by which additional median fins can be added to the body; the first is characteristic of basal euteleosts, while the latter is characteristic of the acanthomorphs. As with the analysis focusing on the full dataset, the angles and lengths of the vectors suggest coordinated patterns of loss and duplication among fins. Again, the presence of additional dorsal and anal fins appears coordinated. The presence of a median ventral fin and of an adipose fin also seems to be coordinated. However, as opposed to the results from the full dataset, here the acute angles between vectors representing fin losses suggest coordinated losses that affect all median and paired fins at once.

In sarcopterygians, the first two dimensions of the MCA (Fig. 3f) explain 55.1 and 27.0% of the variation, respectively. The disparity in fin configurations is limited to the number of dorsal fins and the presence/absence of the dorsal, anal, and caudal fins. Pectoral and pelvic fins are always present in sarcopterygians. The first axis contrasts fishes bearing a caudal, an anal, and two dorsal fins to forms that have lost all of their median fins. Essentially, this contrasts most piscine sarcopterygians to tetrapods. The second axis contrasts forms that have a single dorsal fin and no anal fin to forms that have either two dorsal fins, or where the dorsal and the caudal fins are both absent. This contrasts the derived condition found in dipnoans to a combination of fin characters that is a composite of the other sarcopterygians. As opposed to the results for most of the other analyses, the angles and relative lengths of the vectors from this analysis do not suggest any particularly strong relationships between any of the variables.

MCAs were also performed for the complete gnathostome dataset (125 orders) and the complete osteichthyan dataset (80 orders) (Additional file 3: A–C). The overwhelming actinopterygian pattern was pervasive in both of these analyses, particularly for the osteichthyan dataset. This is not unexpected, since the ordinal diversity and morphological disparity in actinopterygians by far exceed that of the other gnathostome groups.

Fisher’s exact test was used to identify sets of fins that display non-random patterns in their presence/absence, which would be congruent with a modular organization. In other words, if two fins are part of the same fin module, we expect that coordination in their character states (presence, absence, or duplication) should be more frequently observed. Spearman’s rank correlation was used to determine if the significant relationships between pairs of fins revealed by Fisher’s exact test is due to covariation in the presence/absence data, or from antagonistic relationships (one fin is present while the other is absent). In the analysis where unique fin combinations were identified for each individual order, a number of pairs of fins showed non-random patterns in their co-occurrence (Tables 2 and 3). Highly significant results were obtained for the following fin pairs using Fisher’s exact test: median ventral/pectoral, ventrolateral paired/pectoral, pectoral/pelvic, pectoral/dorsal, pectoral/anal, pectoral/caudal, pelvic/dorsal, pelvic/adipose, pelvic/anal, pelvic/caudal, and dorsal/anal fins. All of these pairs of fins were shown to be concurrently present or absent, with the exception of the median ventral/pectoral and the ventrolateral paired/pectoral fins, which display opposite trends in their presence/absence data. Significant results were obtained for the median ventral/ventrolateral paired, median ventral/pelvic, dorsal/adipose, dorsal/caudal, anal/adipose, and anal/caudal fins. The median ventral/ventrolateral paired, dorsal/caudal, anal/adipose, and anal/caudal fins co-occur or are jointly absent, whereas the median ventral/pelvic, ventrolateral paired/pectoral, and dorsal/adipose fins vary in opposite directions.

**Table 2:** *P* values of Fisher’s exact test between fins

	Median ventral fin	Ventrolateral paired fins	Pectoral fins	Pelvic fins	Dorsal fins	Adipose fin	Anal fins
Ventrolateral paired fins	0.022						
Pectoral fins	4.53e-04	0.007					
Pelvic fins	0.016	0.354	2.2e-16				
Dorsal fins	0.235	0.255	3.50e-09	9.41e-08			
Adipose fin	0.052	1	0.083	0.002	0.036		
Anal fins	0.172	1	7.94e-09	0.005	2.01e-12	0.021	
Caudal fin	1	0.607	0.007	2.00e-04	0.013	0.382	0.011

The dataset comprises all possible fin configurations for each order and includes rows with missing data

**Table 3:** Spearman’s rank correlation coefficients between fins (below diagonal) and associated *P* values (above diagonal)

	Median ventral fin	Ventrolateral paired fins	Pectoral fins	Pelvic fins	Dorsal fins	Adipose fin	Anal fins	Caudal fin
Median ventral fin		0.002	<0.0001	0.008	0.087	0.019	0.047	0.583
Ventrolateral paired fins	**0.18**		0.002	0.265	0.942	0.451	0.957	0.271
Pectoral fins	**−0.24**	**−0.18**		<0.0001	<0.0001	0.071	<0.0001	0.002
Pelvic fins	**−0.16**	−0.07	**0.57**		<0.0001	0.003	0.002	<0.0001
Dorsal fins	−0.10	0.00	**0.36**	**0.34**		0.194	<0.0001	0.001
Adipose fin	**0.14**	−0.04	0.11	**0.17**	−0.08		0.027	0.171
Anal fins	**−0.12**	0.00	**0.37**	**0.18**	**0.28**	**0.13**		0.002
Caudal fin	0.03	0.07	**0.18**	**0.23**	**0.19**	0.08	**0.19**	

The dataset comprises all possible fin configurations for each order and includes rows with missing data. Sample sizes vary between 281 and 293 among pairwise comparisons because of missing data. Significant results are in **boldface**

The simplification of the dataset to identify every possible fin combination resulted in 51 unique combinations out of a total of 768 possibilities. Fisher’s exact test (Table 4) identified a single pair of fins, the pectoral/pelvic fins, as highly statistically significant (*P* = 2.16e-05). The pectoral and pelvic fins were shown to be concurrent in their presence or absence by the results of Spearman’s rank correlations (Table 5). Using this dataset, the dorsal/anal fins pair was not found to be statistically significant (*P* = 0.140). Both the pectoral/ventrolateral paired fins (*P* = 0.082) and the pelvic/adipose fins (*P* = 0.085) are marginally non-significant statistically. Based on Spearman’s rank correlations, the pectoral and ventrolateral paired fins were found to vary in opposite directions, whereas the pelvic and adipose fins showed coordination in their presence/absence.

**Table 4:** *P* values of Fisher’s exact test between types of fins

	Median ventral fin	Ventrolateral paired fins	Pectoral fins	Pelvic fins	Dorsal fins	Adipose fin	Anal fins
Ventrolateral paired fins	0.592						
Pectoral fins	0.249	0.082					
Pelvic fins	0.488	0.436	2.16e-05				
Dorsal fins	0.762	0.828	0.300	0.259			
Adipose fin	0.449	1	0.544	0.085	0.810		
Anal fins	1	0.798	0.142	0.206	0.140	0382	
Caudal fin	0.417	0.170	0.750	1	0.775	0.561	0.430

The dataset comprises each unique combination of character states within the entire dataset. Rows with missing data were excluded

**Table 5:** Spearman’s rank correlation coefficients between types of fins (below diagonal) and associated *P* values (above diagonal)

	Median ventral fin	Ventrolateral paired fins	Pectoral fins	Pelvic fins	Dorsal fins	Adipose fin	Anal fins	Caudal fin
Median ventral fin		0.425	0.168	0.445	0.376	0.473	0.589	0.285
Ventrolateral paired fins	0.11		0.032	0.354	0.312	0.486	0.724	0.099
Pectoral fins	−0.20	**−0.30**		<0.0001	0.069	0.195	0.050	0.700
Pelvic fins	−0.11	−0.13	**0.59**		0.083	0.050	0.076	0.931
Dorsal fins	−0.13	−0.14	0.26	0.25		0.474	0.095	0.446
Adipose fin	0.10	−0.10	0.18	0.28	−0.10		0.250	0.306
Anal fins	−0.08	0.05	0.28	0.25	0.24	0.16		0.160
Caudal fin	0.15	0.23	−0.06	−0.01	0.11	0.15	0.20	

The dataset comprises each unique combination of character states within the entire dataset. Rows with missing data were excluded (*N* = 51). Significant results are in **boldface**

## Discussion

For the first time, we have an integrative picture of the evolution of fin configurations and covariation patterns of these appendages among a large diversity of lower vertebrates. The two objectives of this paper were (1) to examine the morphological disparity in fin configurations among basal vertebrates and gain insight into the sequential appearance of median and paired fins in fishes, and (2) to investigate macroevolutionary patterns of co-occurrence among some of the fins, which could then be interpreted as evolutionary modules. These two objectives are not independent. The evolutionary emergence of novel fins could involve the duplication or co-option of pre-existing fin modules. Such scenarios have already been proposed, whether or not explicitly, in the context of the evolution of paired fins in early vertebrates [6, 41], the pectoral and pelvic fins in gnathostomes [17], the spine-brush-complex in symmoriiform sharks [110], the adipose fin in euteleosts [5, 103, 111], and the spinous dorsal fin in acanthomorphs [39]. Modularity also promotes functional and morphological disparity, because modules can be individually optimized without affecting other parts of an organism [22, 32, 112, 113]. Thus, a modular organization of appendages is useful to explain the disparity of fin configurations in fishes, but also at a larger scale of limbs in all vertebrates. The paired appendages of tetrapods provide a very telling example: the fore- and hindlimbs can be modified independently, which was a necessary prerequisite for the evolution of specialized structures, such as the wings in birds or bats [17, 46].

### Disparity in fin configurations

The mapping of fin characters on the supertree reveals which groups are the most disparate in their fin configurations: agnathans, chondrichthyans, and derived actinopterygians display the greatest disparity in fin configurations, although they differ as to which fins are responsible for generating this disparity. Among agnathans, new fins are sequentially added and long ribbon-like fins are gradually modified into more spatially constricted median and paired fins. Thus, the disparity in this part of the tree seemingly results from tinkering with fin configurations and building towards the gnathostome Baüplan. In chondrichthyans, the most important source of disparity is the loss of some (occasionally all) of the median fins. The most disparate fin combinations are found among teleosteans, owing to frequent losses affecting median and/or paired fins, additions of novel fins, or duplications of pre-existing fins.

In agnathans, all of the fins (with the exception of the adipose and pelvic fins that are absent) participate in the observed patterns of disparity in fin configurations. Much of this disparity can be accounted for by the gradual modification of long-based median and paired finfolds into shorter-based dorsal, anal, and pectoral fins. By gradual modifications, we mean that it is seemingly a period where the morphospace is explored, resulting in various combinations of shorter- and longer-based median and paired fins. For instance, shorter-based paired fins appear to have evolved multiple times independently (e.g., *Rhyncholepis* and *Kerreralepis* among birkeniid anaspids; *Lanarkia*, *Phlebolepis*, *Turinia*, and *Shielia* among thelodonts) before the emergence of true pectoral fins as can be found among osteostracans. Absence of the caudal fin also stands out as a source of disparity, yet this is restricted to a few species of hagfishes and lampreys. Among these, two extinct species, the putative hagfish *Gilpichthys greenei* and the putative lamprey *Pipiscius zangerli*, might in fact represent larval organisms [114, 115]. As such, the specimens assigned to these two taxa might not represent adult morphologies, and the scoring of characters could have differed in metamorphosed specimens. In extant species, the caudal fin is generally present, although it can be vestigial or even absent (e.g., *Myxine formosana* [116, 117]). As for the paired fins, ventrolateral paired fins are variably present among anaspids and thelodonts, while shorter-based paired fins that have a position reminiscent of gnathostome pectoral fins are found in some thelodonts and in the osteostracans.

The disparity in fin configurations that is apparent in the chondrichthyan part of the phylogeny can appear surprising given only the modern forms. Paleozoic chondrichthyans, however, present highly disparate morphologies, comparatively making modern holocephalans and elasmobranchs seem conservative [67, 118–121]. Most of the disparity in fin configurations for chondrichthyans can be accounted for by changes in the number of median fins that are present. The anal fin is lost in representatives of numerous chondrichthyan orders. In contrast, the dorsal fin is lacking only in a few chondrichthyan taxa. Most chondrichthyans have two dorsal fins, although the presence of a single dorsal fin is common. There is also some disparity due to the occasional loss of the caudal fin in some batoids. Batoids are characterized by dorso-ventrally flattened bodies, greatly enlarged pectoral fins, and in many species, a long whip-like tail. Propulsion in most of these forms is achieved through undulations (e.g., most skates and sting rays) or oscillations (e.g., eagle rays) of the widened pectoral fins [122–124], which provides a functional context for the loss of the caudal fin when compared to most other chondrichthyans that use a caudal fin-based propulsion. The paired fins do not account for much of the disparity in fin configurations: absence of the pelvic fins is limited to representatives of a single order of extinct chondrichthyans, the Eugeneodontiformes.

In derived actinopterygians, an important part of the disparity in fin configurations relates to the presence/absence of the pelvic fins and to the number of dorsal fins. Pectoral fins are lost far less frequently than the pelvic fins. For instance in teleosteans, the loss of pelvic fins has been reported in more than 100 families belonging to 20 different orders [125], whereas the loss of pectoral fins is reported for only eight teleostean orders in our dataset. The more frequent loss of the pelvic fins could reflect their lesser functional importance for swimming, when compared to the pectoral fins [125–128]. Although this study used a chondrichthyan model, experiments on fin amputations performed on the smooth dogfish (*Mustelus canis*) had shown that the sharks were able to correct for the loss of the pelvic fins using their median and pectoral fins [127]. In contrast, Standen [129] showed that in the rainbow trout (*Oncorhynchus mykiss*), the pelvic fins accomplished complex motions, indicating that their functional importance might have been underestimated. From an eco-morphological perspective, loss of the pelvic fins is often seen in fishes that possess an elongated body shape and occupy complex habitats such as coral reefs or crevices [125, 130, 131]. In these elongated fishes, the pectoral fins are often reduced as well, while the median fins are expanded in length and confluent with the caudal fin [131]. In tetrapods, limb reduction and body elongation are often associated with fossorial or semi-fossorial organisms [132–134]. Thus, in structure-rich habitats, the presence of paired lateral appendages could be disadvantageous, particularly for burrowers or parasitic fishes [135]. Additionally, some of these elongated fishes use anguilliform locomotion, which involves undulations along the entire body length and less emphasis on the use of the paired fins for propulsion [128, 136]. From a macroevolutionary perspective, another hypothesis to explain that the pelvic fins are more frequently lost than the pectoral fins is that pelvic fins appeared after the pectoral fins during the evolutionary history of fishes [11, 20], although an alternative hypothesis has been proposed whereby the pelvic fins appeared first among the anaspids [109]. Additionally, from a developmental perspective, pectoral fins develop prior to the pelvic fins [137, 138], and it has been observed that structures that develop last also tend to be the first to be lost through pedomorphosis [139]. For instance, in reptiles and lissamphibians, patterns of limb reduction reflect developmental sequences: the digits that develop last are the first to be lost in species with reduced limbs [105, 132].

The pelvic fins are frequently lost independently from the pectoral fins: this is observed in at least some representatives of two placoderm orders, one chondrichthyan order and 26 actinopterygian orders. The converse is rare, however. Among piscine gnathostomes, pectoral fin loss is restricted to actinopterygian taxa, and in seven of the eight orders where the pectoral fins are occasionally lost, the pelvic fins also tend to be absent. In fact, the loss of pectoral fins independently from the pelvic fins is only observed in some Stomiidae (Stomiiformes) and Pleuronectiformes. In stomiiform genera where this condition is observed, pectoral fins are present in larvae but are subsequently lost in juveniles and adults [140–145]. In Pleuronectiformes, some species lose their pectoral fins on a single side, while other species lose their pectoral fins on both sides; as with Stomiiformes, this loss takes place during larval metamorphosis [146]. This suggests that loss of the pectoral and loss of the pelvic fins are not entirely independent, which would be an expectation for a paired fins evolutionary module.

The dorsal fin is also responsible for a large part of the disparity in fin configuration in derived actinopterygians: there can be one, two, or three separate dorsal fins, and it can also be entirely absent. There is usually a single anal fin, but it can also be lost, and there can occasionally be two anal fins. Similarly to the paired fins, there is evidence for non-independence in the dorsal/anal fin characters: in orders containing species with two anal fins, two or three dorsal fins tend to be present. Another source of disparity in median fin configurations is the adipose fin which is present in many derived actinopterygians. None of the ostariophysan or euteleostan species that have an adipose fin have second (or third) dorsal fins: instead, they generally have a single centrally placed dorsal fin and a posteriorly located anal fin [147]. Conversely, groups that are close relatives but lack an adipose fin tend to have a “fast-start” morphology with posteriorly placed dorsal and anal fins [147, 148].

### Evolutionary history of fish appendages

#### Median fins

Median fins are present even in the earliest vertebrates. The most basal agnathan fishes are equipped with fairly well-developed median fins which include, in most cases, a caudal fin and elongated dorsal and ventral fins. For instance, the median ventral finfolds of myllokunmingiids span almost the full length of their bodies, as do their long sail-like dorsal fins [7–9, 149]. Myxiniformes also often possess long median ventral finfolds, which although sometimes interrupted around the cloaca, are continuous with the caudal fin. These elongated median fins are reminiscent of the median larval finfold observed during the early ontogeny of more advanced fishes [150, 151]. They are also reminiscent of the extensive dorsal and ventral finfolds found in the more basal cephalochordates, which are continuous around the tail, but also around the anterior tip of the notochord [152–154]. The median fins of cephalochordates are further described as being continuous with one of the two paired metapleural folds, but the latter can hardly be considered as fins because they are hollow structures filled with fluid [155, 156].

Even the most basal agnathans have a caudal fin. A caudal fin is absent, however, in *Gilpichthys greenei* and *Pipiscius zangerli*, two Carboniferous fossil fishes. Although these two taxa display clearly chordate characters, their assignment respectively to the Myxiniformes and Petromyzontiformes remains tentative, and both have been interpreted as possible larval organisms [115]. Thus, the absence of a caudal fin could reflect a larval condition. It is also possible that the apparent lack of a caudal fin is merely a taphonomic artifact. This could arguably be the case for *Pipiscius*, which presents a very posteriorly positioned dorsal fin that could certainly be interpreted as a dorsal extension of the caudal fin. Furthermore, only ten specimens were used for the original description [115]. This explanation is however less likely for *Gilpichthys*, its original description being based on more than 100 specimens [115]. Additionally, traces of the eyes, otic capsules, branchial pouches, and gut have been identified in both species, and these structures were shown to be less decay-resistant than the caudal fin [157, 158].

In more advanced agnathans, a long-based preanal finfold is generally absent. Instead, many taxa possess a shorter-based and more posteriorly positioned anal fin. An anal fin is present in all anaspids that are sufficiently known from their postcranial anatomy. Furthermore, a preanal fin and an anal fin never co-occur in agnathans, with the possible exception of two birkeniid anaspids for which a few spines and an anal plate located anteriorly to the anus [159–161] were provisionally interpreted as evidence for a median ventral fin. Most modern hagfishes and lampreys lack an anal fin. However, the presence of a true anal fin has been observed in a few specimens of *Petromyzon marinus* [162, 163] and of *Lampetra planeri* [164], a phenomenon that has been interpreted as a possible atavism [165, 166]. Anal fins have also been described in two Carboniferous lampreys, *Hardistiella montanensis* [167] and *Mayomyzon pieckoensis* [168]. Based on this evidence, Forey [169] suggested that the absence of an anal fin could be a synapomorphy of recent lampreys. Additionally, the Late Carboniferous hagfish *Myxinikela siroka* is described as having dorsal and ventral fins (= anal fin?) that are continuous with the caudal fin, as in *Mayomyzon*, although in his original description, Bardack [170] raised the possibility that *Myxinikela* might be a juvenile. *Myxinikela*, *Hardistiella*, and *Mayomyzon* represent some of the oldest Myxiniformes and Petromyzontiformes for which complete non-larval specimens are known and, combined with the atavistic reappearance of an anal fin in *P. marinus* and *L. planeri*, this suggests that the appearance of an anal fin occurred before the anaspids. Thus, an anal fin could be a plesiomorphic characteristic of vertebrates or even of craniates if the ventral fin of *Myxinikela* is homologous to an anal fin. An anal fin is absent in the oldest fossil lamprey, *Priscomyzon*, but phylogenetic analyses resolve *Mayomyzon* as the most basal petromyzontid, while *Priscomyzon* is more derived [171–173]. As for more crownward taxa, the presence of an anal fin is considered primitive for chondrichthyans, acanthodians, and osteichthyans; its absence in some Paleozoic sharks (e.g., *Cladoselache*, stethacanthids, and symmoriids) is considered as a derived condition [174].

As opposed to the median ventral fin, the long-based dorsal fins of myllokunmingiids are not so rapidly modified into shorter-based dorsal fins. Many agnathan taxa bear short-based and comparatively more posteriorly positioned dorsal fins (Petromyzontiformes, Loganelliiformes, Shieliiformes, Phlebolepidiformes, Furcacaudiformes, Osteostraci), whereas the Jamoytiiformes retain elongated dorsal fins. Long-based dorsal fins also occur in numerous chondrichthyan (e.g., *Pleuracanthus gaudryi*, *Chondrenchelys problematica*) and osteichthyan (e.g., *Regalescus glesne*, *Acanthurus major*) taxa. It is reasonable to assume that the dorsal fin is not constrained in its anterior extent and position, as opposed to the anal fin, which cannot extend anteriorly past the position of the anus. The Gymnotiformes provide a striking example: these fishes have elongated anal fins that extend along the majority of the ventral midline of the body, yet the anus is displaced anteriorly in these forms, positioned under the pectoral fins or even under the head, thus remaining in front of the anterior limit of the anal fin [175–177].

#### Duplications of the dorsal fins

Duplications of the dorsal fin seem to have occurred numerous times independently during the evolutionary history of fishes. Most extant lampreys have two dorsal fins. Among osteostracans, *Ateleaspis*, *Aceraspis*, and *Hirella* possess two dorsal fins and are resolved as basal members of this group [15, 178–181]. Among the most basal orders of placoderms, antiarchs and stensioellids generally possess a single dorsal fin, but the material for brindabellaspids and pseudopetalichthyids precludes interpretation of dorsal fin characters. In other groups of placoderms where dorsal fin characters are known, ptyctodontids have two dorsal fins, whereas rhenanids and arthrodires have a single dorsal fin. Among acanthodians, climatiiforms, diplacanthiforms, and ischnacanthiforms have two dorsal fins. Acanthodiforms possess a single dorsal fin, but this is considered as secondarily derived for this group.

Lund [174] expressed that the plesiomorphic condition for the number of dorsal fins in chondrichthyans could not be determined at the time and could just as well have been a single dorsal fin or two dorsal fins. The most basal articulated undisputed elasmobranchs known from the fossil record, *Doliodus problematicus* and *Antarctilamna prisca*, have anterior dorsal fins, but most of the postcranial region is unknown and thus insufficient to assess the presence of a posterior dorsal fin [182, 183]. Additionally, in the *Antarctilamna* material, a spine with a shallow insertion that had initially been interpreted as a displaced dorsal fin spine is now thought to be a pectoral fin spine, whereas a second type of spine with a deeper insertion is interpreted as a median fin [109, 183, 184]. Furthermore, phylogenies have not reached a stable consensus concerning the interrelationships of basal Euchondrocephali (see, e.g., [185–187]). Our supertree analysis places the iniopterygians as the most basal euchondrocephalan order, although they are resolved as the sister clade to all other chondrichthyans in Lund et al. [186]. Of course, in light of the growing support for the hypothesis that acanthodians are stem chondrichthyans, this would imply that the plesiomorphic condition for the total group chondrichthyans is in fact the presence of two dorsal fins.

Among osteichthyans, the presence of two dorsal fins has been considered as plesiomorphic [188]. *Guiyu oneiros*, resolved as a stem sarcopterygian [60], was originally reconstructed with a single dorsal fin [189], but has recently been reinterpreted as having two dorsal fins [190]. All other sarcopterygians have two dorsal fins, with the exception of a few dipnoans, elpistostegalians, and tetrapods. The Early Devonian *Dialipina* is resolved either as a basal osteichthyan [54, 55, 58, 59, 61, 191–193] or as the most basal actinopterygian [57, 99, 102, 189, 194–197], and it possesses two dorsal fins [99]. Among other non-acanthomorph actinopterygians, a second dorsal fin is also found in a single fossil Ionoscopiformes species [100] and in a few extant Siluriformes belonging to the Plotosidae [1, 198–200]. This suggests that the presence of two dorsal fins would have been lost early during actinopterygian evolution [102], but that this character would have subsequently been reacquired more than once independently.

Acanthomorphs are characterized by the possession of an anterior spinous dorsal fin [201]. In some taxa the spinous and soft dorsal fins are continuous and connected by a fin web, whereas in others they are widely separated. In our scoring of characters, we considered that dorsal fins where the bases were not connected by a fin web constituted separate dorsal fins. The dorsal fin(s) of acanthomorphs can be interpreted in two different ways. One hypothesis is that the acanthopterygian anterior spinous dorsal fin results from a duplication of the posterior soft dorsal fin module [39]. Another hypothesis is that the second or third dorsal fin in acanthopterygians results from the subdivision of an originally more elongated fin [202]. As such, acanthomorphs retain a single dorsal fin which is regionalized, thus giving the impression that there are two (or three) dorsal fins [16]. Our supertree analysis places the Lampridiformes at the base of the acanthomorph radiation. The Aipichthyoidea, resolved as stem Lampridiformes [203, 204], possess a single dorsal fin, for which the anterior portion is generally supported by two to five fin spines [204–207], although there are 12 fin spines in *Homalopagus multispinosus* [207]. In crown Lampridiformes, dorsal fin spines are present in Veliferidae but are considered to have been secondarily lost in other forms [203]. Among other acanthomorph orders that were resolved as the most basal in our supertree analysis, Percopsiformes and Polymixiiformes also possess a single dorsal fin where the leading edge is generally supported by a few spines [1]. In light of this evidence, the hypothesis of a regionalized dorsal fin cannot be ignored.

Taken together, the phylogenetic distribution of dorsal fin conditions suggests that duplications of the dorsal fin occurred multiple times during the evolutionary history of fishes. It also suggests that two dorsal fins might have been the condition for the common ancestor to both osteostracans and gnathostomes. This character would have subsequently been lost and then occasionally reacquired in many fish lineages.

#### Paired fins

**Fig. 4:**
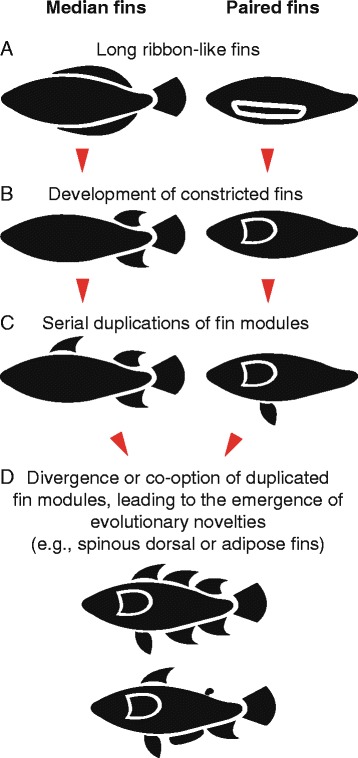
Hypothesized scenario for the evolution of median and paired fins. Both median and paired fins developed first as elongated ribbon-like structures (**a**) that are gradually modified into short-based fins (**b**). Serial duplications of fin modules lead to the emergence of novel fins such as the pelvic fins or a second dorsal fin (**c**). Divergence or co-option of some fin modules also leads to the evolution of novel fins, such as the adipose fin of euteleosts or the spinous dorsal fin of acanthomorphs (**d**)

The first evidence of true paired fins in craniates is in the Anaspidiformes and Jamoytiiformes, generally in the form of long ribbon-like paired folds that are ventrolateral in position. A notable exception can be found in the Myxiniformes, where for a single genus, *Neomyxine*, we tentatively scored for the presence of ventrolateral paired fins. *Neomyxine* possesses paired folds of skin located immediately above the gill openings [208–210]. These skin folds are not used for swimming but rather as support when specimens settle on the substrate [209]. Furthermore, because these structures are located dorsally to the branchial apertures and because *Neomyxine* is not basal relative to other hagfishes, these paired skin folds are unlikely to be homologous to the ventrolateral paired fins found in other agnathans [49, 211], although some thelodonts also have paired fins that are inserted dorsally to the branchial apertures [63]. Thus, excluding *Neomyxine*, ventrolateral paired fins appear with the anaspids and can also be found in some thelodonts. The question regarding the homology of these paired fins has been debated for many years. Some authors consider that true paired fins must be constricted and supported by an endoskeletal girdle and fin radials [47, 48]. An alternative hypothesis is that paired fins evolved first as lateral extensions of the body, and that paired girdles only appeared later during the evolutionary history of basal vertebrates [109, 212]. Shubin et al. [17] proposed an evolutionary scenario whereby (1) paired fins first appeared as elongated ventrolateral expansions along the body wall, (2) these expansions were then modified into shorter-based pectoral appendages only, (3) and later pelvic fins appeared among gnathostomes as serial homologues of the pectoral fins. We find the latter hypothesis reasonable: it would not be surprising that paired and unpaired fins share a similar evolutionary history (Fig. 4) considering the remarkable anatomical and developmental similarities between the paired and unpaired fins [41, 151, 213]. Furthermore, based on gene expression patterns during fin development in lampreys and sharks, it was suggested that the genetic programming associated with median fin development was subsequently redeployed to the lateral mesodermal plate, giving rise to the paired fins [6, 41]. A previous study focusing on gene expression patterns in cephalochordates had similarly led Schubert et al. [214] to hypothesize that part of the developmental programs involved in tail outgrowth in basal chordates could have been co-opted towards paired appendage development in vertebrates. Alternatively, it could be argued that the pelvic fins, and not the pectoral fins appeared first [109], or that paired fins evolved multiple times independently during the evolutionary history of vertebrates [215].

As for short-based paired fins, true pectoral fins are considered to have appeared with the osteostracans, although pituriaspids also have pectoral fenestrae, suggesting that pectoral fins were present in these taxa as well [216]. The pectoral fins of osteostracans are supported by endoskeletal elements and are under muscular control: they can thus be considered as homologous to the pectoral fins of gnathostomes [11, 13, 15]. Additionally, some thelodonts possess muscularized and moveable paired fins that are in a pectoral position [11, 63, 217]. Girdle-supported pelvic fins are absent in agnathans [18, 47, 190, 211] and are first observed among placoderms [16, 18, 19]. Antiarch placoderms have been resolved at the base of the gnathostome diversification in most of the recent phylogenetic studies [54–57, 59, 61] and were thought to be devoid of pelvic fins [65]. However, Zhu et al. [18] recently described a pelvic girdle in *Parayunnanolepis*. Additionally, our supertree analysis places the Pseudopetalichthyida stemward to the antiarch placoderms, making it the most basal gnathostome order. The most well-preserved pseudopetalichthyid articulated material belongs to *Pseudopetalichthys problematica*, which is known to possess both pectoral and pelvic fins [218]. This suggests that the presence of pelvic fins is likely to be plesiomorphic for gnathostomes.

### Evidence for fin evolutionary modules

**Fig. 5:**
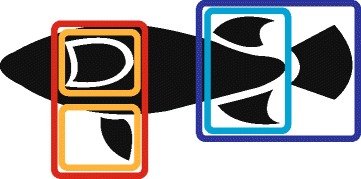
Hypothesized fin modules. The pectoral and pelvic fins form a paired fins evolutionary module that can be dissociated, leading to individualized pectoral and pelvic fin modules. The dorsal and anal fins form a second evolutionary fin module nested within a larger median fins evolutionary module

Based on the mapping of fin characters on the supertree, some pairs of fins are more frequently associated, either through coordinated duplication events or through coordinated losses, which is congruent with hypotheses that together they form evolutionary modules. This is the case for the dorsal and anal fins where the presence of a second anal fin is associated with the presence of a second or third dorsal fin. Mabee et al. [39] suggested that the dorsal and anal fins were linked through the presence of both positioning and patterning modules. Although patterning modules refer to the development of endo- and exoskeletal supports [39, 40, 219], the effect of the patterning module could extend to the resorption of the larval median finfold. More precisely, during fish larval development, there are generally dorsal and ventral median finfolds that are continuous with a caudal finfold [150, 151]. During development, these finfolds are resorbed except in places where dorsal, anal, and caudal fins will develop [151]. Here, we hypothesize that the mechanism underlying duplication of a non-resorption zone of the larval finfold could very well be reflected dorso-ventrally, leading to coordinated duplications of the dorsal and anal fins. This pattern also emerges in their coordinated loss patterns. For instance, in actinopterygians, results from the multiple correspondence analyses suggest that loss of the dorsal, anal, and caudal fins can be coordinated. Likewise, the results also show that loss of the pectoral and pelvic fins can be coordinated. Coordination of fin losses is not limited to actinopterygians: the results of the multiple correspondence analyses for chondrichthyans and sarcopterygians also show coordinated losses of the median fins. Evidence for a dorsal and anal fins evolutionary module has been proposed for lungfish, in light of the observations that, in earlier forms, the dorsal and anal fins present equivalent positions along the antero-posterior body axis, that they have similar morphologies, particularly with respect to fin supports, and that they were coordinately lost at the end of the Devonian [220]. It is unclear, however, if the dorsal and anal fins module suggested in Johanson et al. [220] involves the anterior dorsal fin, the posterior dorsal fin, or both dorsal fins. At a population scale, it was found that in the Arctic charr (*Salvelinus alpinus*), anatomical and developmental patterning of the dorsal and anal fins were highly similar, but differed largely from that of the caudal fin: this was interpreted as supporting the patterning modules proposed by Mabee et al. [39] for the dorsal and anal fins [219, 221]. A patterning module was also hypothesized for the caudal fin [219]. In contrast, in a recent study focusing on variational modularity in two cyprinid species, we showed good support for the hypothesis that the dorsal, anal, and caudal fins formed one variational module including the caudal peduncle, while the paired fins formed another variational module [222]. Because modularity is a hierarchical concept, a hypothesis of evolutionary modularity worth investigating is that the median fin system as a whole could constitute one module, the paired fin system could constitute a second independent module, and the dorsal and anal fins could constitute a third module nested within the median fins module (Fig. 5). Quasi-independent median and paired fin modules would help explain why there is so much disparity in median fin configurations in chondrichthyans, compared to the paired fins, which are largely unaffected.

We have demonstrated that the co-occurrence of some sets of fins is non-random. Among these, the pectoral, pelvic, dorsal, anal, and caudal fins have all been found to be non-independent. This reflects the most common fin combinations found in the dataset where all of these fins co-occur, more specifically, the fin combinations that are characteristic of most actinopterygian orders (single dorsal and anal fins, a caudal fin, pectoral and pelvic fins) and of most chondrichthyan and sarcopterygian orders (two dorsal fins, a single anal fin, a caudal fin, pectoral and pelvic fins). When the analysis focuses on unique fin combinations, only the pectoral/pelvic fins show non-independence. The strong relationship between the pectoral and pelvic fins is concurrent with hypotheses that they form a paired fins evolutionary module.

A relationship was also found between the adipose fin and the median ventral fin. The adipose fin is considered as an evolutionary novelty among teleostean taxa and might also constitute a new fin module [103, 110]. An adipose fin evolved at least twice independently, once within the Otophysi and a second time in the Euteleostei [5, 103]. Development of the adipose fin is known to differ between otophysans and euteleosteans, supporting the hypothesis of multiple independent origins: in Characiformes, the adipose fin appears as an outgrowth following the complete resorption of the larval finfold, while in Salmoniformes, it develops as a remnant of the larval finfold [5]. As for the median ventral fin, the positive relationship with the adipose fin stems from a few euteleostean families that, in addition to the adipose fin, possess a rayless finfold in front of the anal fin that is often described as a ventral adipose fin (Retropinnidae, Stomiidae, Paralepididae). However, a similar ventral fin is also found in at least one family, the Sundasalangidae (Clupeiformes), prior to the appearance of the adipose fin, as well as in euteleostean families that do not have adipose fins (Phallostethidae, Hypoptychidae). As opposed to the adipose fin which has been the object of numerous recent investigations [5, 103, 111, 147, 148], to our knowledge no work has focused on the origin or homology of the so-called ventral adipose fin. Developmental and histological work would be necessary to establish if this median ventral fin is homologous among these taxa.

A dorsal-anal fin module is well supported by developmental data [40–42, 219, 221, 223]. It has also been inferred based on the similarities in the relative positioning of these two fins across species [39]. Because the positioning module inferred by Mabee et al. [39] has been identified at a macroevolutionary scale, it qualifies as an evolutionary module. Herein, we provide further evidence for a dorsal-anal fin evolutionary module, with indications that its effect also extends to the coordinated losses and duplications of these fins in different species.

Co-occurrence of the pectoral and pelvic fins is extremely well supported in our analyses. Both paleontological and embryological studies support the idea that the pelvic fins could have originated by a duplication of the pectoral fins module [20, 21, 224, 225]. Based on this hypothesis, it follows that the co-occurrence of these two fins would be expected. An alternative possibility is that the pectoral and pelvic fin modules have dissociated and become independent modules during the evolutionary history of fishes [10, 20, 45, 46, 226]. As evidence for this latter hypothesis, Coates and Cohn [10] mentioned that there is no example in which the pelvic appendages are a direct copy or identical serial homologues of the pectoral fins. Additionally, some primitive gnathostomes (e.g., *Parayunnanolepis*, *Kathemacanthus*, *Lupopsyrus*, *Brochoadmones*, *Cheirolepis*) have pectoral and pelvic fins that are both anatomically different and positionally decoupled [109]. One could argue, however, that the paired fins present extremely similar morphologies in chimaerids (C. Riley and E. Grogan, personal communication; R. Cloutier, personal observation) and in many sarcopterygians [188, 190]. Furthermore, biserial fin designs evolved convergently in pectoral and pelvic fins in some chondrichthyan and sarcopterygian taxa, as did uniserial fin designs in osteolepiforms [105]. Considering that independent loss of the pectoral or pelvic fins occurs almost only in actinopterygians, perhaps the dissociation of the paired fins module is a generalized characteristic for this group, which was independently acquired in eugeneodontiform sharks.

## Conclusions

Although the sequential emergence of fins among fishes has been discussed on empirical grounds, the results from this analysis support a longstanding idea that both the median and paired fins would have appeared first as long-based or ribbon-like structures, before being modified into more spatially constricted appendages. Additionally, for the first time, we have a quantified picture of the covariation in fin presence at a large phylogenetic scale. Our results highlight that even with a dataset comprising semi-quantitative characters, there is compelling evidence that the pectoral and pelvic fins, and the dorsal and anal fins form two distinct evolutionary modules. The results also suggest an interesting hypothesis whereby the dorsal/anal fins module could be nested within a larger median fins module. Combined with the results from our previous analysis on variational modularity in cyprinids [222], this suggests that patterns of morphological integration and modularity that are identified within populations can translate into integration at a macroevolutionary scale. An important next step will be to validate this hypothesis using fully quantitative methods, as well as to investigate the consequences of these putative evolutionary modules on patterns of morphological disparity. Because the hypotheses of modularity that we are testing are largely based on evidence from developmental data, this would provide a striking example linking developmental to variational and evolutionary modules.

## Additional files

Additional file 1: Source trees and additional results of the supertree analyses. A: List of source trees that were incorporated into the MRP supertree analysis. B: 50% majority rule consensus of the supertrees obtained using Nixon’s Parsimony Ratchet. C: Strict consensus of the supertrees obtained using Nixon’s Parsimony Ratchet. D: Supertree obtained using optimum parsimony. E: Insights of the supertree into basal vertebrate interrelationships. (PDF 510 kb)

Additional file 2: A CSV file containing fin characters that were mapped on the supertree and analyzed. (CSV 28 kb)

Additional file 3: Additional results of the multiple correspondence analyses focusing on gnathostomes and osteichthyans. A: Biplot of the first two dimensions of the MCA focusing on gnathostomes. B: Biplot of the first two dimensions of the MCA focusing on osteichthyans. C: Description of additional multiple correspondence analyses results. (PDF 217 kb)
