# Supplementary material for: Fin modules: an evolutionary perspective on appendage disparity in basal vertebrates
Source: BMC Biol. 2017 Apr 27;15:32. doi: 10.1186/s12915-017-0370-x (PMC5406925; doi:10.1186/s12915-017-0370-x)
Supplement: Supplementary file 1 — Source trees and additional results of the supertree analyses. A: List of source trees that were incorporated into the MRP supertree analysis. B: 50% majority rule consensus of the supertrees obtained using Nixon’s Parsimony Ratchet. C: Strict consensus of the supertrees obtained using Nixon’s Parsimony Ratchet. D: Supertree obtained using optimum parsimony. E: Insights of the supertree into basal vertebrate interrelationships. (PDF 510 kb) [file 12915_2017_370_MOESM1_ESM.pdf]

Fin modules: An evolutionary perspective on appendage disparity in basal vertebrates

Olivier Larouche, Miriam L Zelditch and Richard Cloutier

Additional file 1.

A: List of source trees that were incorporated into the MRP supertree analyses.

Tree sections	Published phylogenies
Agnathans	[8, 48-53, 149, 171-173, 227-231]
Placoderms and Acanthodians	[54-59, 61, 193, 232-237]
Chondrichthyans	[185-187, 238-258]
Actinopterygians	[99, 201, 204, 259-293]
Sarcopterygians	[45, 68, 108, 189, 191, 195, 294-310]

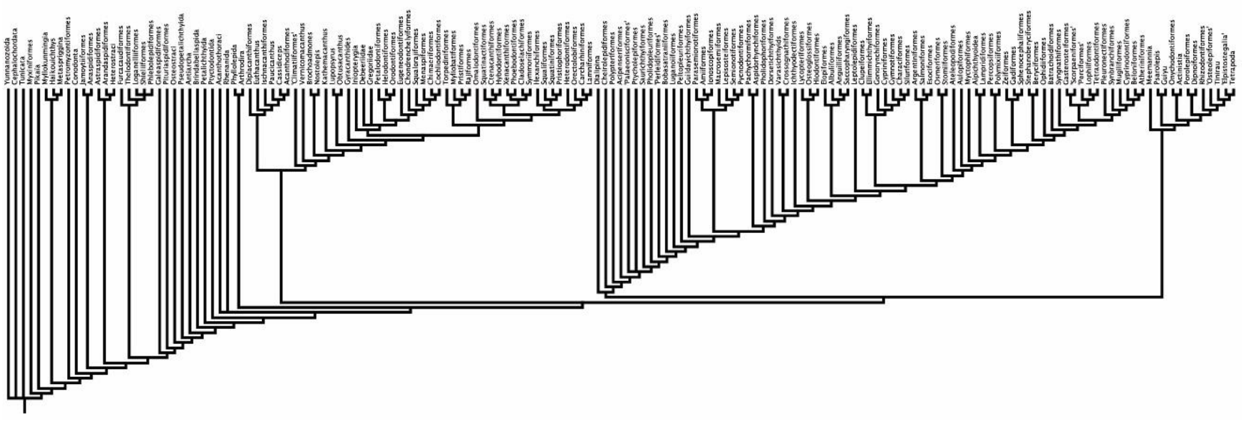

B: 50% majority rule consensus of the supertrees obtained using Nixon's parsimony ratchet.

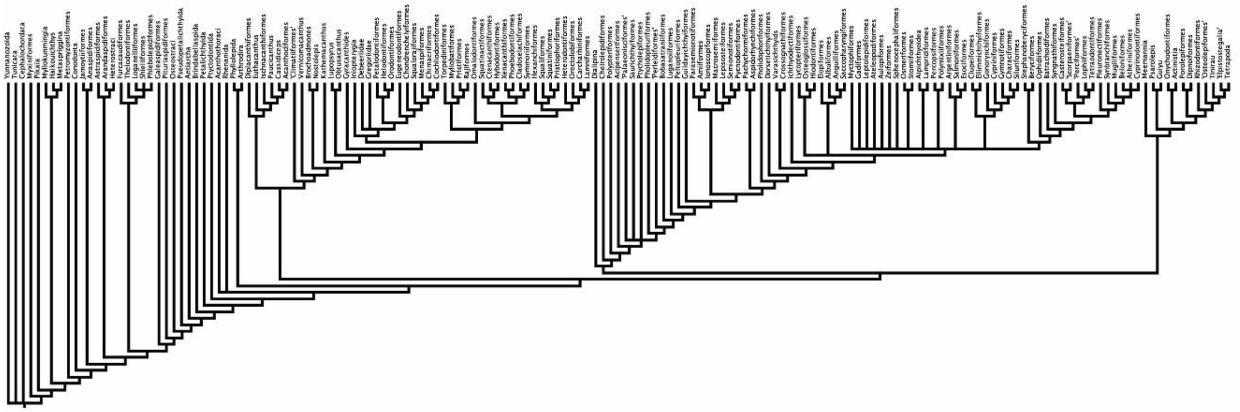

C: Strict consensus of the supertrees obtained using Nixon’s parsimony ratchet.

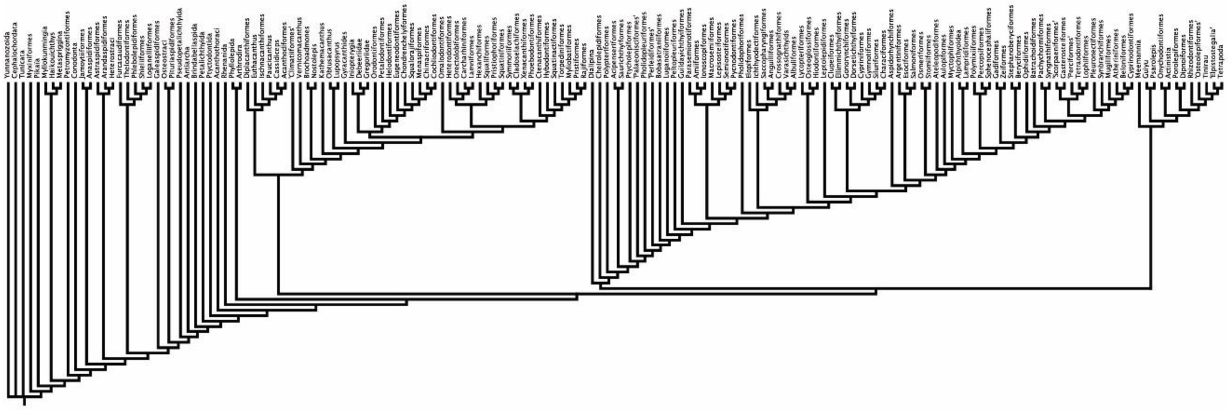

D: Supertree obtained using optimum parsimony.

E: Insights of the supertree into basal vertebrate interrelationships

The phylogenetic relationships of basal vertebrates have not yet reached a stable consensus. Four groups that have been strongly debated concerning their interrelationships to other fishes are the conodonts, the extant agnathans (hagfishes and lampreys), the placoderms and the acanthodians. The consensus topology from our supertree considers cyclostomes, acanthodians and placoderms as paraphyletic groups. Furthermore, the supertree places conodonts within vertebrates, placoderms as stem gnathostomes, and acanthodians as stem chondrichthyans.

Conodont interrelationships have been vigorously debated, the two prevailing views being that they are either stem chordates [52, 311-315], or that they are stem craniates or even stem vertebrates [49, 316-324]. Morphological arguments that have been used to promote the craniate or vertebrate hypothesis include the presence of a ray-supported caudal fin, eyes with extrinsic

musculature and paired sensory structures that are interpreted as indirect evidence of a brain [49, 165, 319, 321-323]. Arguments against the vertebrate interpretation of conodonts include that cephalisation is low in conodonts, that their trunk musculature is V-shaped rather than W-shaped, and that they lack dermal fin rays supporting the fins, a gill basket and a dermal skeleton [52, 315]. Additionally, based on histological investigations, some authors have hypothesized that conodonts possess vertebrate-like hard tissues in the form of globular calcified cartilage, cellular bone, an enamel-homologue and dentine [316, 325-327], but these findings have been met with criticisms [52, 315, 328-333]. Ultimately, the position of conodonts in phylogenies results largely from how some of the homologies are interpreted [52, 334]. Although the conodont fossil record is extremely abundant, it mostly consists of elements of the oral apparatus and only a few articulated specimens with postcranial material have been discovered. As with the cyclostomes, the debate over conodont interrelationships remains an open question and will probably remain so until further well-preserved material is discovered.

Two competing hypotheses have been proposed concerning the interrelationships of extant agnathans and gnathostomes: (1) either lampreys and hagfishes form a clade called the cyclostomes [335-337], (2) or hagfishes are craniates while lampreys are vertebrates, making the "cyclostomes" paraphyletic relative to gnathostomes [13, 16, 169, 178, 211, 338-342]. Although cyclostome monophyly was initially proposed based on morphological arguments [e.g. 337, 343], more recently it has been advocated mainly on the grounds of molecular phylogenetic analyses [e.g. 344, 345-349], whereas the analyses based on morphological datasets and incorporating fossil taxa have generally resolved hagfishes and lampreys as paraphyletic relative to gnathostomes [12, 48-50, 52, 171, 350, 351]. Although our consensus topology considers the cyclostomes as paraphyletic, the debate seems far from settled as both hypotheses are difficult to reconcile on the grounds of both morphological and molecular evolution. However, cyclostome monophyly implies either that lampreys and gnathostomes share an impressive amount of homoplastic characters, or that there is a no-less impressive history of reversals among hagfish characters [165, 348, 349]. We agree with the opinion that cyclostome monophyly cannot be resolved on the basis of molecular data alone and requires the incorporation of evidence from the fossil record [351, 352].

For many years, placoderms and acanthodians were considered by most to be monophyletic groups: placoderms were considered as the sister group to crown gnathostomes [353, 354], while acanthodians were considered as a clade of stem osteichthyans [353, 355, 356]. Although some

studies have focused on intra-relationships within placoderms [232, 234, 354, 357] or acanthodians [233, 235, 236], recent matrix-based analyses at a larger phylogenetic scale do not support the monophyly of either of these groups. The accumulated evidence now seems to support the prevailing view that placoderms are paraphyletic stem gnathostomes, while acanthodians are either paraphyletic stem chondrichthyans [56-60, 192], or even polyphyletic, with some taxa as stem gnathostomes, others as stem chondrichthyans and yet others as stem osteichthyans [54, 55]. A single recent analysis has resolved acanthodians as the monophyletic sister group of chondrichthyans [61]. Importantly, all of these recent phylogenetic analyses that have focused on the interrelationships of placoderms, acanthodians and gnathostomes used modified data matrices from Brazeau [54]. In some of these analyses, it has been observed that the addition or exclusion of taxa and/or characters reveal conflicting signals within the dataset as well as unstable topologies [55, 358]. Our consensus supertree results in a topology where placoderms are paraphyletic stem gnathostomes and acanthodians are paraphyletic stem chondrichthyans, although we realize that this reflects the most common findings based on multiple analyses that are not independent from one another. We conclude that the monophyly of these groups is unlikely, but cannot be entirely rejected. Lists of characters have been proposed as supporting the monophyly of both placoderms [65, 354, 359] and acanthodians [235].

Among crown gnathostomes, the supertree analysis recovered most ordinal groupings that have generally been recognized as clades (e.g., Euchondrocephali, Elasmobranchii, Squalomorphii, Galeomorphii, Osteoglossomorpha, Elopomorpha, Otocephala, Acanthomorpha, Tetrapodomorpha). A notable exception occurred when performing the analysis for chondrichthyans. In a first run of the supertree analysis with batoids represented as four separate orders, the resulting trees showed an unusual topology where the batoids were polyphyletic and variously distributed among Paleozoic elasmobranchs. We subsequently ran the analysis with "Batoidea" as a terminal branch, thus enforcing the known monophyly of the group, and obtained the topology presented in Fig. 2, where batoids are placed as the most basal of the Elasmobranchii. Although some have suggested that batoids are highly derived selachians [e.g., 239, 241, 360, 361], a more recent view which is well supported by molecular-based phylogenies is that batoids are an elasmobranch clade that shares a common ancestor with the Selachii [245, 253, 257, 362, 363]. Yet in this case, the supertree approach failed to retrieve this topology. The inconsistency in the placement of the batoids can likely be imparted to two issues in our dataset

of phylogenetic trees. The first issue is described as the “rogue branch” problem in Ragan [72], whereby a single branch appears at radically different places in the trees considered. The second issue stems from the fact that our chondrichthyan supertree analysis incorporated source trees focusing on fossil taxa and source trees focusing on extant taxa, and that there is minimal overlap in the taxa representing the basal part of the elasmobranchs’ phylogeny among these source trees. Most phylogenetic analyses that aim to resolve fossil chondrichthyan interrelationships incorporate both euchondrocephalan and elasmobranch taxa. However, batoids are almost never incorporated in these analyses. In contrast, batoids are often incorporated in phylogenetic analyses that aim to resolve extant chondrichthyan interrelationships, whereas Paleozoic elasmobranchs are excluded. This lack of overlap among source trees in the basal part of the elasmobranchs phylogeny results in the Paleozoic elasmobranchs being resolved as stem euselachians, whereas the batoids are resolved as the most basal elasmobranchs instead of being placed among neoselachians. Batoids are most likely pulled further towards the stem because chimaeras are incorporated in analyses focusing on the interrelationships of extant chondrichthyans, and because in these analyses batoids are most frequently resolved as basal to all other neoselachians.
