# Supplementary material for: Fin modules: an evolutionary perspective on appendage disparity in basal vertebrates
Source: BMC Biol. 2017 Apr 27;15:32. doi: 10.1186/s12915-017-0370-x (PMC5406925; doi:10.1186/s12915-017-0370-x)
Supplement: Supplementary file 3 — Additional results of the multiple correspondence analyses focusing on gnathostomes and osteichthyans. A: Biplot of the first two dimensions of the MCA focusing on gnathostomes. B: Biplot of the first two dimensions of the MCA focusing on osteichthyans. C: Description of additional multiple correspondence analyses results. (PDF 217 kb) [file 12915_2017_370_MOESM3_ESM.pdf]

Fin modules: An evolutionary perspective on appendage disparity in basal vertebrates

Olivier Larouche, Miriam L Zelditch and Richard Cloutier

Additional file 3

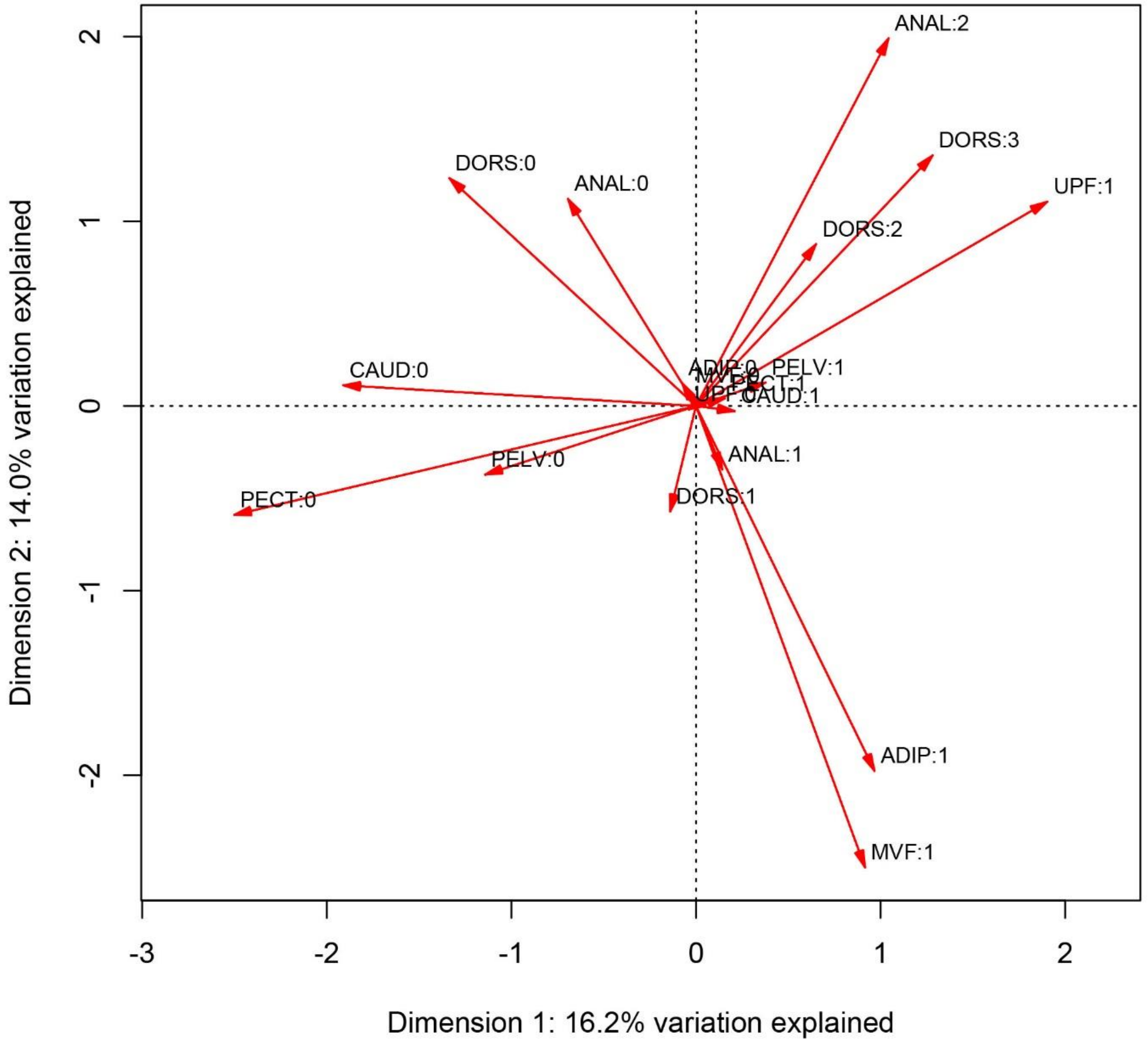

A: Biplot of the first two dimensions of the MCA focusing on gnathostomes.

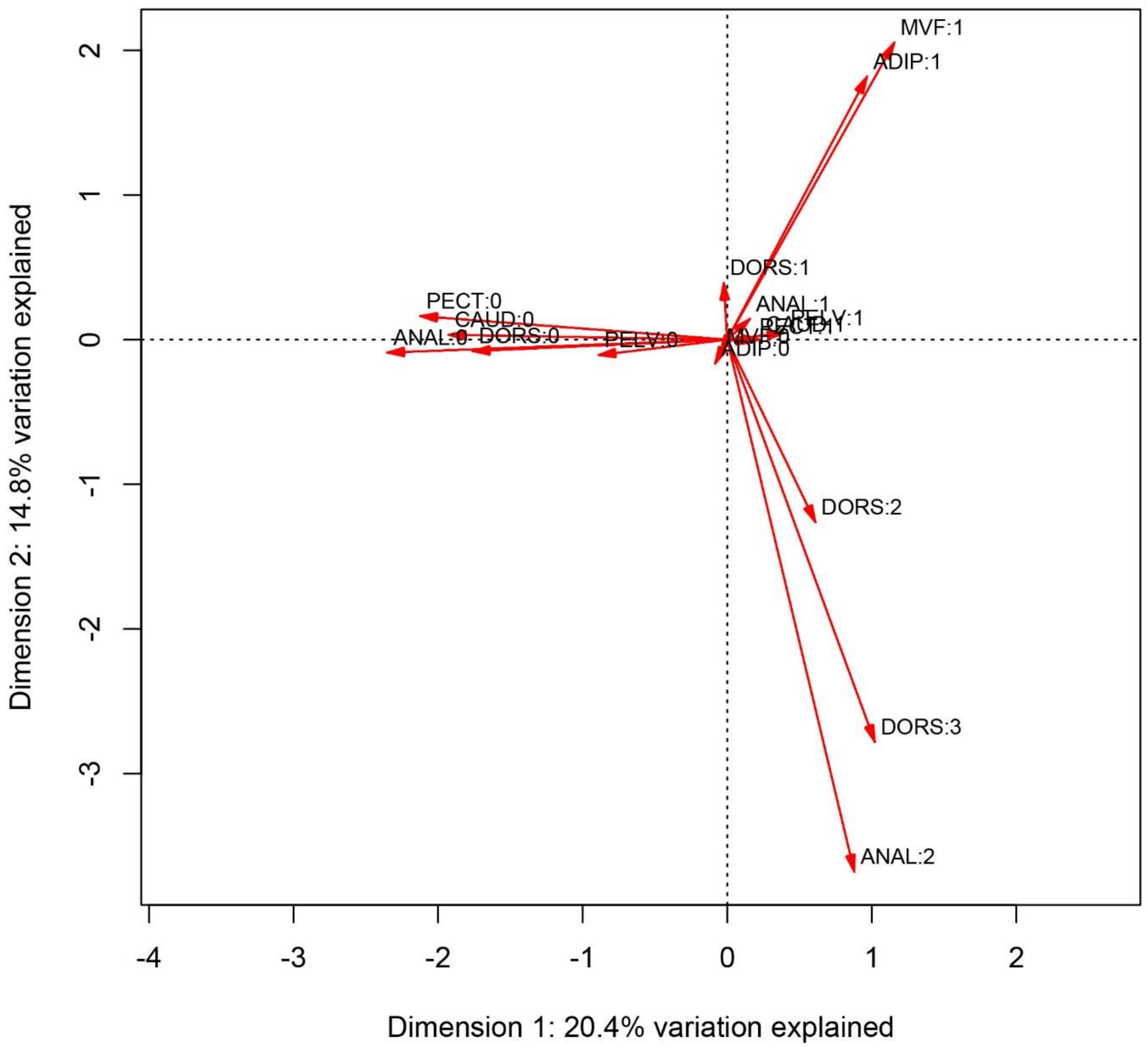

B: Biplot of the first two dimensions of the MCA focusing on osteichthyans.

C: Description of additional multiple correspondence analyses results

Supplementary analyses were conducted for the total group gnathostomes and osteichthyans. These yielded results similar to those for the actinopterygian clade, differing only in the percentage of variation explained by the major axes. Multiple correspondence analyses could not be performed for placoderms alone because of a lack of variation in fin characters. In placoderms,

only two of the fins show variation: the pelvic fins, which are occasionally absent, and the dorsal fins, which can be absent, present in a single copy or duplicated.

The first two dimensions of the analysis focusing on gnathostomes explain 16.2% and 14.0% of the total variation in fin configurations, respectively. The patterns obtained for the actinopterygians are pervasive in these first two dimensions of the analysis performed on gnathostomes, although the angles between the vectors are wider. The first dimension of the MCA opposes fishes characterized by the loss of all fins to those that have either two or three dorsal and anal fins, or additional sets of fins in the form of unconstricted paired fins, median ventral fins, or adipose fins. The second dimension opposes forms having the dorsal and anal fins either absent, duplicated, or triplicated to fishes that have median ventral fins and/or adipose fins. In the analysis focusing on osteichthyans, the results are qualitatively identical as the analysis focusing on actinopterygians, although the first two dimensions of the MCA now explain 20.4% and 14.8% of the variation, respectively.
